# Phase II study of ipilimumab monotherapy in Japanese patients with advanced melanoma

**DOI:** 10.1007/s00280-015-2873-x

**Published:** 2015-09-26

**Authors:** N. Yamazaki, Y. Kiyohara, H. Uhara, S. Fukushima, H. Uchi, N. Shibagaki, A. Tsutsumida, S. Yoshikawa, R. Okuyama, Y. Ito, T. Tokudome

**Affiliations:** Department of Dermatologic Oncology, National Cancer Center Hospital, Tokyo, Japan; Dermatology Division, Shizuoka Cancer Center, Shizuoka, Japan; Department of Dermatology, Shinshu University School of Medicine, Matsumoto, Japan; Department of Dermatology and Plastic Surgery, Faculty of Life Sciences, Kumamoto University, Kumamoto, Japan; Department of Dermatology, Graduate School of Medical Sciences, Kyushu University, Fukuoka, Japan; Department of Dermatology, University of Yamanashi Hospital, Yamanashi, Japan; Research and Development, Bristol-Myers K.K., 6-5-1, Nishishinjuku, Shinjuku-ku, Tokyo 1631328 Japan

**Keywords:** Ipilimumab, Immune-checkpoint inhibitor, Melanoma, Phase II study, Japanese patients

## Abstract

**Purpose:**

Ipilimumab is designed to block cytotoxic T-lymphocyte antigen-4 to augment antitumor T cell responses. In studies of predominantly Caucasian patients with advanced melanoma, ipilimumab was associated with durable response, long-term survival benefit, and a manageable safety profile. This phase II study assessed the safety of ipilimumab in Japanese patients with unresectable stage III or IV melanoma.

**Methods:**

Patients received ipilimumab 3 mg/kg every 3 weeks for four doses. The database lock for the original analysis was in August 2014. Overall survival, progression-free survival, and data on deaths were based on an updated, follow-up analysis (database lock April 2015).

**Results:**

Data are reported from 20 patients. Fifteen patients (75 %) received all four doses of ipilimumab during induction. Twelve patients (60 %) had at least one drug-related adverse event (AE), and no patients discontinued due to a drug-related AE. There were no deaths related to study drug. The most common drug-related AEs were rash (*n* = 7), pyrexia (*n* = 3), increased aspartate aminotransferase (AST; *n* = 3), and increased alanine aminotransferase (ALT; *n* = 3). Twelve patients (60 %) reported immune-related AEs (irAEs); most frequent were skin (*n* = 9) and liver (*n* = 3) disorders. Grade 3 irAEs were ALT and AST elevation (*n* = 2) and diabetes mellitus (*n* = 1). Two patients had a partial response and two had stable disease, yielding a 20 % disease control rate. Median overall survival and progression-free survival were 8.71 and 2.74 months, respectively.

**Conclusion:**

Ipilimumab 3 mg/kg had a manageable AE profile in this Japanese patient population with clinical outcomes similar to that in Caucasian patients.

**ClinicalTrials.gov identifier:**

NCT01990859.

**Electronic supplementary material:**

The online version of this article (doi:10.1007/s00280-015-2873-x) contains supplementary material, which is available to authorized users.

## Introduction

Ipilimumab is a fully human IgG_1_ monoclonal antibody that blocks cytotoxic T-lymphocyte-associated antigen-4 (CTLA-4), a key negative regulator of endogenous T cell-mediated immune responses. Blockade of CTLA-4 has been shown to result in effector T cell activation, proliferation, and infiltration into tumors, as well as functional suppression and depletion of regulatory T cells, which enhance antitumor immunity, resulting in tumor cell death [1–7]. Ipilimumab has been approved at a dose of 3 mg/kg by regulatory agencies in the USA and over 40 other countries for the treatment of advanced (unresectable or metastatic) melanoma [7]. Approval was based upon a significant overall survival (OS) benefit demonstrated in two phase III studies [8, 9]. A 3-year survival rate of 22 % was observed in a pooled analysis of 1861 ipilimumab-treated patients with advanced melanoma from ten prospective clinical trials and two retrospective studies. The analysis showed a plateau in the survival curve beginning at approximately 3 years, with follow-up to 10 years in some patients [10].

In one phase III study, ipilimumab significantly improved OS in previously treated patients with advanced melanoma compared with a melanoma glycoprotein 100 vaccine [8]. Similarly, in a phase III study in treatment-naïve patients with advanced melanoma, ipilimumab at 10 mg/kg in combination with dacarbazine significantly increased OS compared with dacarbazine alone [9]. Most adverse events (AEs) experienced with ipilimumab are inflammatory in nature and may reflect ipilimumab’s immune-based mechanism of action [8, 9, 11–13]. These immune-related AEs (irAEs) are generally manageable using product-specific treatment guidelines, which recommend appropriate monitoring and corticosteroid treatment, and, if necessary, treatment interruption or discontinuation [11, 13–15].

Data from a phase I study of ipilimumab plus chemotherapy in Japanese patients with advanced non-small cell lung cancer showed that the efficacy and safety of ipilimumab were similar to those observed in studies of non-Japanese patients [16]. The primary objective of the current phase II study was to evaluate the safety of ipilimumab monotherapy at 3 mg/kg in Japanese patients with advanced melanoma.

## Materials and methods

### Patients

Japanese patients who were 20 years of age or older were eligible for enrollment if they had histologically or cytologically confirmed diagnosis of malignant melanoma, untreated or previously treated, stage III (unresectable) or stage IV melanoma. Other inclusion criteria included measurable/evaluable disease per modified World Health Organization (mWHO) criteria within 28 days of first dose of study drug; a life expectancy of at least 16 weeks; an Eastern Cooperative Oncology Group (ECOG) performance status of 0 or 1; adequate hematologic, renal, and hepatic function; and negative screening tests for human immunodeficiency virus, hepatitis B, and hepatitis C and human T-lymphotropic virus type 1. Prior adjuvant melanoma therapy was permitted. Key exclusion criteria included primary ocular or mucosal melanoma; active brain metastases with symptoms or requiring corticosteroid treatment (patients with stable, asymptomatic, controlled brain metastases were permitted if corticosteroids were not required for management); a history of, or current, autoimmune diseases; and prior anticancer therapy <4 weeks prior to first study treatment.

### Study oversight

This study was sponsored by Bristol-Myers K.K. The study protocol was approved by the Institutional Review Board at each study institute, and research was conducted in accordance with the standards specified by Article 14, Paragraph 3, and Article 80-2 of the Pharmaceutical Affairs Law, and Good Clinical Practice, as defined by the Ministerial Ordinance Concerning the Standards for the Implementation of Clinical Studies on Pharmaceutical Products and concerning notifications, and in accordance with ethical principles underlying the Declaration of Helsinki. All study participants provided written informed consent prior to enrollment.

### Study design and treatment

**Fig. 1:**
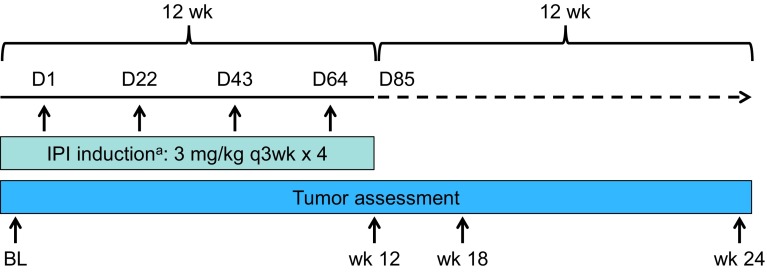
Study design. *BL* baseline, *D* day, *IPI* ipilimumab, *PD* progressive disease, *q3wk* every 3 weeks, *wk* week. ^a^Patients with PD, intolerability toxicity or who discontinued study treatment during induction entered the follow-up phase and were followed for safety and survival for ≥1 year after the last patient’s first treatment

This was a single-arm, open-label study of ipilimumab monotherapy 3 mg/kg in untreated or previously treated Japanese patients with unresectable or metastatic melanoma (ClinicalTrials.gov, NCT01990859) (Fig. 1). Patients received four doses of ipilimumab 3 mg/kg intravenously every 3 weeks for a total of 12 weeks. Patients with disease progression, intolerable toxicity, or who discontinued study treatment in the induction phase, entered a follow-up phase and were followed for safety and survival for at least 1 year after the last patient’s first treatment. The planned study sample size was approximately 18 treated patients.

### Study endpoints and assessments

The primary objective of this study was to assess the safety of ipilimumab monotherapy in Japanese patients with advanced melanoma. To assess safety, AEs were graded according the National Cancer Institute Common Terminology Criteria for Adverse Events (CTCAE), version 3.0. irAEs were also summarized. Guidelines for the management of AEs were provided by the sponsor (Supplemental Fig. 1) and have been published previously [11].

The secondary objective was to explore the antitumor activity [best overall response rate (BORR): complete response (CR) plus partial response (PR)] of ipilimumab monotherapy using mWHO criteria. Tumor assessments were performed at screening; weeks 12, 18, and 24; and every 12 weeks thereafter. Determination of a response required confirmation with a subsequent scan at least 4 weeks later.

Exploratory objectives included the assessment of disease control rate [DCR; CR plus PR plus stable disease (SD) assessed using mWHO criteria], OS, progression-free survival (PFS), and antidrug antibody (ADA) response to ipilimumab. Blood samples for assessment of ADAs were drawn before the ipilimumab infusion at weeks 1, 4, 7, and 10 and at the end of treatment. Samples were analyzed at an external laboratory (Pharmaceutical Product Development, LLC, Richmond, Virginia, USA).

Safety and efficacy were evaluated for all treated patients. The original database lock for safety and efficacy outcomes was in August 2014. These analyses were based on patients with at least 90 days of follow-up after the last dose of drug. OS, PFS and data on deaths are based on a follow-up analysis (database lock April 2015) 1 year after the last patient received the last dose of ipilimumab. BORR and DCR were calculated along with corresponding two-sided 95 % confidence intervals (CIs). OS and PFS were calculated using Kaplan–Meier estimates, with medians and corresponding two-sided 95 % CIs reported.

## Results

### Patients and treatment

A total of 26 patients were enrolled into this study at six centers in Japan between December 2013 and January 2014; 20 patients were treated with ipilimumab, of whom 16 (80 %) had received prior anticancer therapy for advanced disease and 4 (20 %) were previously untreated. Six patients were enrolled, but not treated (five no longer met study criteria, and one withdrew consent). Patient demographics are shown in Table 1. At study entry, the majority had M1c disease (70 %), were ECOG performance status 0 (70 %), and had elevated lactate dehydrogenase (LDH) levels (60 %). Treated patients received a median of four cycles of ipilimumab, with 15 patients (75 %) receiving all four doses.

**Table 1:** Patient demographics

Characteristic	Treated patients (*N* = 20)
Gender, male [*n* (%)]	10 (50)
Race, Japanese [*n* (%)]	20 (100)
Age, years [median (range)]	62.5 (29–76)
M stage at study entry [*n* (%)]	
M0	1 (5)
M1a	1 (5)
M1b	4 (20)
M1c	14 (70)
ECOG performance status [*n* (%)]	
0	14 (70)
1	6 (30)
Baseline LDH [*n* (%)]	
Normal	8 (40)
Elevated	12 (60)
Prior systemic anticancer therapy [*n* (%)]	
Yes	16 (80)
No	4 (20)

*ECOG* Eastern Cooperative Oncology Group, *LDH* lactate dehydrogenase

### Safety

Safety data are summarized in Table 2. All patients reported at least one AE, and nine patients (45 %) had AEs of grade 3/4 in severity. Twelve patients (60 %) had drug-related AEs, of which 3 (15 %) were grade 3 in severity [increased alanine aminotransferase (ALT), increased aspartate aminotransferase (AST), and diabetes mellitus]; there were no grade 4 drug-related AEs. The most frequently reported drug-related AE was rash. Eleven patients reported a serious AE (SAE); the events were considered drug-related in three patients (grade 3 ALT/grade 2 AST increase/grade 2 C-reactive protein increase in one patient, grade 2 AST/ALT increase in one patient, and grade 3 diabetes mellitus in one patient). No patient discontinued the study due to toxicity related to study drug.

**Table 2:** Ipilimumab safety data summary

	Treated patients (*N* = 20)	
	Any grade	Grade 3 or 4^a^
AEs [*n* (%)]		
Any AE	20 (100)	9 (45)
Drug-related serious AEs	3 (15)	2 (10)
Treatment-related AEs	12 (60)	3 (15)

	Any grade occurring in ≥ 2 patients	Grade 3
Treatment-related AEs^a,b^		
Rash	7 (35)	0 (0)
Pruritus	2 (10)	0 (0)
Pyrexia	3 (15)	0 (0)
ALT increased	3 (15)	1 (5)
AST increased	3 (15)	1 (5)
Decreased appetite	2 (10)	0 (0)
Diarrhea	2 (10)	0 (0)
Diabetes mellitus	–	1 (5)

*AE* adverse event, *ALT* alanine aminotransferase, *AST* aspartate aminotransferase

^a^AEs were graded according to the National Cancer Institute Common Terminology Criteria for Adverse Events, version 3.0

^b^According to the most recent version of the Medical Dictionary for Regulatory Activities

Twelve patients (60 %) had an irAE; the most frequently reported occurred in the skin and liver (Table 3). Other irAEs were gastrointestinal disorders, immune system disorders, or metabolism and nutrition disorders. Most irAEs were grade 1/2 in severity. Three patients had a grade 3 irAE: ALT elevation (*n* = 1), AST elevation (*n* = 1), and diabetes mellitus (*n* = 1). There were no grade 4 irAEs and no gastrointestinal perforations or neurological irAEs. The irAEs reported as SAEs were elevations in ALT (grade 2) and AST (grade 2) in one patient, elevations in ALT (grade 3) and AST (grade 2) in one patient, and diabetes mellitus (grade 3) in one patient.

**Table 3:** irAEs occurring in patients receiving ipilimumab^a,b^

MedDRA system organ class preferred term [*n* (%)]	Grade 1	Grade 2	Grade 3	Any grade
Any irAE	5 (25)	4 (20)	3 (15)	12 (60)
Skin and subcutaneous tissue disorders	5 (25)	4 (20)	0 (0)	9 (45)
Rash	4 (20)	3 (15)	0 (0)	7 (35)
Pruritus	1 (5)	1 (5)	0 (0)	2 (10)
Alopecia	1 (5)	0 (0)	0 (0)	1 (5)
Investigations	1 (5)	0 (0)	2 (10)	3 (15)
ALT increased	1 (5)	1 (5)	1 (5)	3 (15)
AST increased	1 (5)	1 (5)	1 (5)	3 (15)
Blood bilirubin increased	0 (0)	1 (5)	0 (0)	1 (5)
Gastrointestinal disorders	1 (5)	1 (5)	0 (0)	2 (10)
Diarrhea	1 (5)	1 (5)	0 (0)	2 (10)
Immune system disorders	1 (5)	0 (0)	0 (0)	1 (5)
Hypersensitivity	1 (5)	0 (0)	0 (0)	1 (5)
Metabolism and nutrition disorders	0 (0)	0 (0)	1 (5)	1 (5)
Diabetes mellitus	0 (0)	0 (0)	1 (5)	1 (5)

^a^Patients may have had more than one event

^b^No grade 4 or 5 events were reported

*ALT* alanine aminotransferase, *AST* aspartate aminotransferase, *irAE* immune-related adverse event, *MedDRA* Medical Dictionary for Regulatory Activities

The time to onset from first dose of ipilimumab for grade 2 or higher on-study irAEs was 1.1 weeks (grade 2 hepatic irAE) to 13.6 weeks (grade 3 diabetes). For those events that resolved, the time to resolution ranged from 0.6 weeks (grade 3 hepatic irAE) to 14.0 weeks (grade 2 skin irAE). Eleven out of the 12 patients who had on-study irAEs received steroids for irAE management. All grade 2 or higher on-study irAEs resolved to grade 1 or less following administration of steroids or symptomatic therapies, except for the grade 3 diabetes event. The grade 3 diabetes mellitus irAE remained unresolved at the time of reporting, and the patient was receiving insulin therapy and other antidiabetic medications.

Adverse events that occurred more than 90 days after the last dose of ipilimumab included 2 endocrine irAEs [grade 1 hypothyroidism and grade 2 hypopituitarism (also reported as an SAE)] and 1 skin irAE (grade 1 vitiligo). There were 13 deaths during the study, all due to disease progression (five deaths occurred within 90 days of the last dose of study drug and eight occurred more than 90 days after the last dose of study drug).

### Antitumor activity

**Fig. 2:**
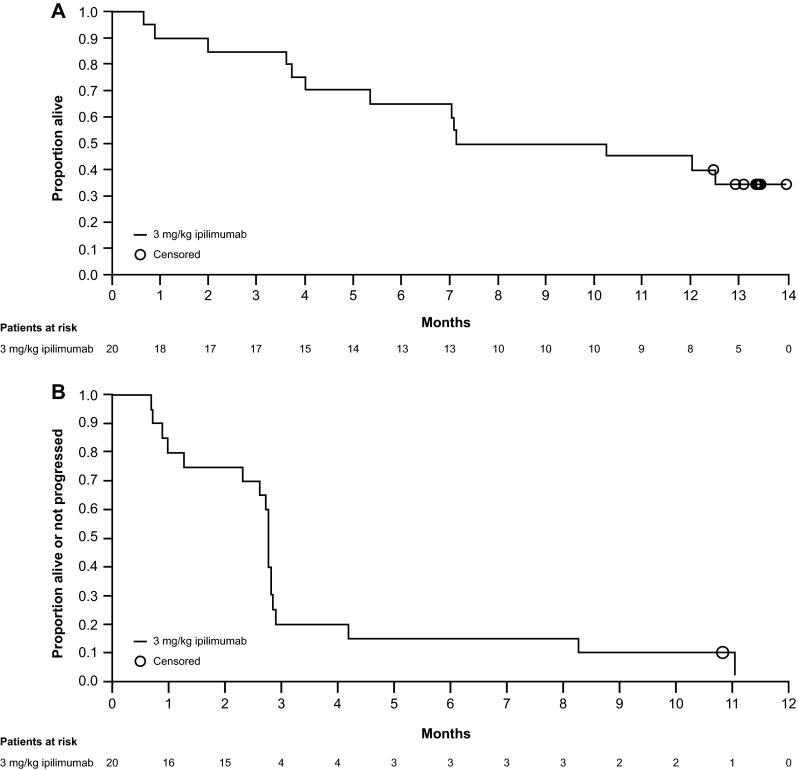
Kaplan–Meier curves for overall survival (**a**) and progression-free survival (**b**)

At 12 weeks after the last patient’s first treatment, four patients demonstrated antitumor activity (Table 4). Two patients (10 %) (1 pretreated, 1 untreated) had PR, and two patients (10 %) (both previously treated) had SD. The remaining patients had either progressive disease [13 (65 %)] or were not evaluable [3 (15 %)] due to death before week 12. Disease control rate was 20 % (95 % CI 5.7–43.7), and BORR was 10 % (95 % CI 1.2–31.7). The median OS was 8.71 months (95 % CI 3.71–not reached), and the median PFS was 2.74 months (95 % CI 1.25–2.83) (Fig. 2).

**Table 4:** Best overall response and disease control rates

Best overall response	Treated patients (*N* = 20)
Complete response (CR) [*n* (%)]	0 (0)
Partial response (PR) [*n* (%)]	2 (10.0)
Stable disease (SD) [*n* (%)]	2 (10.0)
Progressive disease [*n* (%)]	13 (65.0)
Not evaluable [*n* (%)]	3 (15.0)
Best overall response rate [*n* (% [95 % CI])^a^]	2 (10 [1.2–31.7])
Disease control rate [*n* (% [95 % CI])^b^]	4 (20 [5.7–43.7])

*CI* confidence interval

^a^Number of patients with CR or PR/number of treated patients

^b^Number of patients with CR, PR, or SD/number of treated patients

### Pharmacokinetics

The study also investigated serum concentrations of ipilimumab relative to time of dosing. Results in Japanese patients were generally similar to those previously reported in predominantly Caucasian populations (data not shown).

### Immunogenicity data

Three samples from two patients were confirmed positive for ADAs. Both patients had received all four doses of ipilimumab without dose delay and neither had any peri-infusional hypersensitivity or anaphylactic reactions. One of the ADA-positive patients had SD, and the other had progressive disease. None of the three samples tested developed neutralizing antibodies.

## Discussion

The findings from this study showed that the immune-checkpoint inhibitor ipilimumab, administered at a dose of 3 mg/kg every 3 weeks for four doses, had a manageable safety profile with evidence of antitumor activity in this group of Japanese patients with advanced melanoma. The safety profile was consistent with the previous experience with 3 mg/kg ipilimumab in global clinical studies involving primarily Caucasian patients [8, 9]. As reported previously, the most common treatment-related AEs with ipilimumab therapy were immune-related, presumably reflecting the drug’s immune-based mechanism of action, affecting the skin, liver, gastrointestinal tract, and endocrine system [12]. Most of the irAEs observed in the current study were mild-to-moderate in severity, and no grade 4 irAEs were reported. The tolerability of ipilimumab in the current study is supported by the observation that no patient withdrew from the study due to a drug-related AE. Importantly, no new safety signals were observed in this group of Japanese patients compared with those seen in earlier studies across the world [8, 9], and there were no study-drug-related deaths.

Extensive global experience with ipilimumab enabled the development of effective treatment guidelines for the management of irAEs (Supplementary Fig. 1; [11]). The guidelines emphasize early diagnosis and appropriate treatment as essential to minimize severe and potentially life-threatening complications [11, 13]. These guidelines were used as a basis for the development of the protocol for the current study, and following these guidelines may have contributed to the absence of the grade 4 irAEs. Ipilimumab treatment guidelines recommend symptomatic treatment for mild irAEs with delay or omission of a dose and intense monitoring for more severe events. In the case of persistent or high-grade irAEs, ipilimumab should be discontinued and patients should be treated with high-dose systemic corticosteroids (with slow taper to reduce the risk of relapse). Patients who are refractory to steroid treatment may require alternative immunosuppressive therapies, such as infliximab (antitumor necrosis alpha treatment). Clinical studies have shown that most irAEs are reversible when these guidelines are followed [11, 13].

In the current study, most of the patients with on-study irAEs received steroids. All irAEs except the grade 3 diabetes mellitus resolved to grade 1 or less, including the two patients with grade 3 liver irAEs. Both of these patients resumed ipilimumab treatment and completed the full four doses of ipilimumab without recurrence of the liver irAE. The grade 3 diabetes was ongoing but manageable with insulin at the time of reporting. The patient had no medical history of diabetes, suggesting it was caused by an immune-mediated mechanism. However, previous global experience with ipilimumab suggests diabetes and hyperglycemia are uncommon irAEs [11]. In the current study, most irAEs resolved within 1 to 14 weeks; this is consistent with previous global experience where the time to resolution for the most common irAEs typically ranged from 4 to 9 weeks depending on the organ system involved [13].

The majority of irAEs in the current study occurred during ipilimumab treatment or within 90 days of the last dose. However, two endocrine irAEs (grade 1 hypothyroidism and grade 2 hypopituitarism) and one skin irAE (grade 1 vitiligo) were reported more than 90 days after the last dose. Grade 2 hypopituitarism was managed with steroid therapy, and at the time of database lock, the event remained unresolved as treatment with hormone replacement therapy continued. No treatment was required for grade 1 hypothyroidism and vitiligo. These findings are consistent with previous reports that ipilimumab may cause late-onset irAEs, including endocrine-related events [17]. For example, in the international phase III trial MDX010-20, one patient (4.2 %) who received ipilimumab plus placebo experienced late-onset (>70 days after last dose) grade 1 vitiligo and grade 2 hypothyroidism [17]. Patients treated with ipilimumab plus gp100 in MDX010-20 also reported late-onset AEs including grade 1 vitiligo (*n* = 3, 5.6 %), grade 2 hypogonadism and reduced serum testosterone (*n* = 1, 1.9 %), and grade 1 diarrhea, grade 2 proctitis, and grade 3 colitis (*n* = 1, 1.9 %) [17]. These data emphasize the importance of ongoing patient follow-up and vigilance for irAEs after treatment has stopped.

Assessment of antitumor activity with ipilimumab was a secondary objective in this study. The BORR of 10 % and DCR of 20 % are similar to those reported in the phase III study with ipilimumab 3 mg/kg in previously treated, primarily Caucasian patients [8]. Additionally, as reported in other studies, responses were observed in patients regardless of whether they had received prior therapies [8, 9]. Long-term follow-up of ipilimumab-treated patients in global clinical studies has shown the potential of ipilimumab for durable long-term survival in approximately 20 % patients with advanced melanoma [10]. This durable survival benefit, despite relatively low response rates, is likely a result of ipilimumab’s immune-mediated mechanism of action, with which it can take longer for a detectable antitumor response to occur compared with cytotoxic agents. The kinetics of antitumor activity may also be different from other agents, meaning that conventional response criteria (such as those of mWHO) may not fully capture the antitumor activity of ipilimumab [13].

Although ipilimumab is a fully human antibody, data suggest the potential for the induction of ADAs [18]. In the current study, two patients were positive for ADAs. However, because of the low number of positive samples throughout the study and the observed safety and activity profiles, particularly in the patients who were positive for ADAs (neither had a peri-infusional hypersensitivity or anaphylactic reaction, and one patient had SD), the development of ADAs after ipilimumab treatment is not expected to have a clinically relevant impact on safety or activity [18].

Antitumor activity has been demonstrated with another immune-checkpoint inhibitor, nivolumab, approved in Japan in 2014 for patients with unresectable or metastatic melanoma regardless of prior treatment. Nivolumab is a fully human IgG4 programmed death 1 (PD-1) immune-checkpoint inhibitor with a different mechanism of action from that of ipilimumab [19]. In a phase III trial (CheckMate 037 [20]), 405 patients with advanced melanoma who had progressed after receiving ipilimumab, or ipilimumab and a BRAF inhibitor if they were BRAF V600 mutation-positive, were treated with nivolumab monotherapy or chemotherapy. Nivolumab treatment resulted in a confirmed objective response rate (ORR) of 32 % that was significantly higher than that in the chemotherapy group (11 %). Safety profiles with nivolumab were similar to those of previously untreated patients, suggesting that prior treatment with ipilimumab did not adversely affect patient safety. A phase II trial (CheckMate 069 [21]) of treatment-naïve patients demonstrated that combination therapy with ipilimumab and nivolumab resulted in significantly improved ORR compared with ipilimumab monotherapy (61 % compared with 11 %, respectively, in BRAF wild-type patients). A phase III study of combination treatment (CheckMate 067) reported an ORR of 44 % with nivolumab, 58 % with nivolumab plus ipilimumab, and 19 % with ipilimumab alone [22]. Median PFS was 6.9, 11.5, and 2.9 months, respectively [22].

In summary, findings from the current phase II study showed that ipilimumab monotherapy at 3 mg/kg had a manageable safety profile and produced antitumor activity in this Japanese patient population, regardless of whether the patient had received prior treatment. These findings are consistent with the known safety and activity profile of ipilimumab established in global clinical studies.

## Electronic supplementary material

Supplementary material 1 (DOCX 488 kb)
