# Supplementary material for: Phase II study of ipilimumab monotherapy in Japanese patients with advanced melanoma
Source: Cancer Chemother Pharmacol. 2015 Sep 26;76(5):997–1004. doi: 10.1007/s00280-015-2873-x (PMC4612321; doi:10.1007/s00280-015-2873-x)
Supplement: Supplementary file 1 — Supplementary material 1 (DOCX 488 kb) [file 280_2015_2873_MOESM1_ESM.docx]

Article type: Original article

**Phase II study of ipilimumab monotherapy in Japanese patients with advanced melanoma**

N. Yamazaki • Y. Kiyohara • H. Uhara • S. Fukushima • H. Uchi • N. Shibagaki • A. Tsutsumida • S. Yoshikawa • R. Okuyama • Y. Ito • T. Tokudome

N. Yamazaki • A. Tsutsumida
Department of Dermatologic Oncology, National Cancer Center Hospital, Tokyo, Japan

Y. Kiyohara • S. Yoshikawa
Dermatology Division, Shizuoka Cancer Center, Shizuoka, Japan

H. Uhara • R. Okuyama
Department of Dermatology, Shinshu University School of Medicine, Matsumoto, Japan

S. Fukushima
Department of Dermatology and Plastic Surgery, Faculty of Life Sciences, Kumamoto University, Kumamoto, Japan

H. Uchi
Department of Dermatology, Graduate School of Medical Sciences, Kyushu University, Fukuoka, Japan

N. Shibagaki
Department of Dermatology, University of Yamanashi Hospital, Yamanashi, Japan

Y. Ito • T. Tokudome
Research and Development, Bristol-Myers K.K., Tokyo, Japan

**Correspondence to:**

Takuto Tokudome, Research and Development, Bristol-Myers K.K., Tokyo, 6-5-1, Nishishinjuku, Shinjuku-ku, Tokyo 1631328, Japan

**Supplementary Fig. 1** Toxicology management algorithms^a^

1. Gastrointestinal toxicity management algorithm

1. Hepatotoxicity management algorithm

1. Skin toxicity management algorithm

1. Endocrinopathy management algorithm

^a^Source: Bristol-Myers Squibb Company (2014) YERVOY^®^ (ipilimumab) investigator brochure
